# Double VEGF/HGF Gene Therapy in Critical Limb Ischemia Complicated by Diabetes Mellitus

**DOI:** 10.1007/s12265-020-10066-9

**Published:** 2020-09-01

**Authors:** Piotr Barć, Maciej Antkiewicz, Barbara Śliwa, Katarzyna Frączkowska, Maciej Guziński, Tomasz Dawiskiba, Małgorzata Małodobra-Mazur, Wojciech Witkiewicz, Diana Kupczyńska, Bartłomiej Strzelec, Dariusz Janczak, Jan Paweł Skóra

**Affiliations:** 1 Department and Clinic of Vascular, General and Transplantation Surgery, Jan Mikulicz-Radecki Medical University Hospital, Wroclaw Medical University, Wroclaw, Poland; 2 Department of Radiology, Jan Mikulicz-Radecki Medical University Hospital, Wroclaw Medical University, Wroclaw, Poland; 3 Molecular Techniques Unit, Wroclaw Medical University, Wroclaw, Poland; 4 Regional Specialized Hospital in Wroclaw, Research and Development Center, Wroclaw, Poland

**Keywords:** Gene therapy, VEGF, HGF, Critical limb ischemia, Diabetes mellitus

## Abstract

Critical leg ischemia (CLI) complicated by diabetes mellitus (DM), which is a very common and dangerous disease, represents the ultimate stage of peripheral arterial disease. Patients are treated with antiplatelet drugs, statins and limb revascularization, but a significant number of patients are not candidate for revascularization. Literature shows that in such cases, gene therapy could be a perfect therapeutic option. The aim of our study was to evaluate efficacy of double vascular endothelial growth factor/hepatocyte growth factor (VEGF/HGF) gene therapy in patients with CLI complicated by DM. We observed that 90 days after administration, serum level of VEGF and ankle-brachial index increased significantly (*p* < 0.001) and rest pain decreased significantly compared with the control group (*p* < 0.002). Moreover considerable improvement in vascularization was observed in computed tomography angiography (*P* = 0.04). Based on the results of this study, we suggest that the therapy with pIRES/VEGF165/HGF bicistronic plasmid administration is a safe and effective method of treatment of patients with both CLI and DM.

**Figure:**
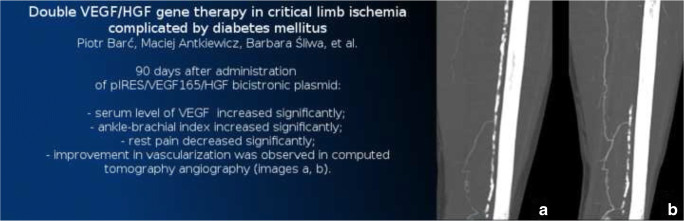
Graphical abstract

Graphical abstract

## Introduction

Critical leg ischemia (CLI) indicates the final stage of peripheral arterial disease (PAD). Due to the fact that CLI is most commonly caused by atherosclerosis obliterans, it is heavily associated with smoking and diabetes mellitus (DM) [5, 6, 17]. PAD leads to approximately 500–1000 new cases of CLI per million people per year and it touches men three times more often than women [1–3, 5, 6, 17]. Revascularization is a cornerstone of therapy to prevent limb amputation, and despite advance in both open and endovascular surgery, many patients are doomed to leg dismemberment. The facts that about one-third of people suffering from CLI in United States are poor or no-option patients and that 30% of CLI patients have to undergo leg amputation with mortality rate of 20% or higher show that this disease makes serious problem nowadays [3–7, 13, 17]. Because there is no directly CLI aimed medication, the only cure for “not candidate for revascularisation” (NCR) patients is oral antiplatelet drugs, statins, treatment of DM and smoking cessation. Nevertheless, this patients’ cohort has a particularly poor prognosis, including a 1 year amputation rate of 40% and a mortality as high as 20% [4–6]. Salvation is offered by therapeutic angiogenesis, which by administration of angiogenic molecules directly to damaged tissues leads to formation of new vessels. Angiogenic gene therapy trials were seen a few years ago as a promising emerging therapy for different types of ischemia. We found many studies testing gene therapy in treatment of various diseases caused by ischemia with miscellaneous outcomes [8, 9, 12–14, 16, 21, 28]. Many of angiogenic factors such as vascular endothelial growth factor (VEGF), fibroblast growth factors (FGF1 and FGF2), or hepatocyte growth factor (HGF) were tested in clinical trials. HGF provides the best results but is still not sufficient alone [2, 9, 20, 25]. Also, VEGF encoding plasmid seemed to be inefficient in single therapy [9, 12, 14, 16, 27]. The poor outcome of therapy aimed on administration of only one angiogenic factor is probably caused by the fact that the angiogenic process requires coordinated expression of multiple genes [9, 10, 12, 14, 20]. However, VEGF is the key factor in angiogenesis by stimulating the proliferation and migration of endothelial cells, which leads to the formation of new vessels. This process also requires the presence of other growth factors such as HGF [2, 10, 20, 25, 27]. The HGF receptor is present on various types of cells including endothelium [2, 20]. Some studies report that presence of HGF in extracellular matrix multiplies the activity of VEGF during angiogenesis [2, 20, 25]. Unfortunately, protein forms of angiogenic factor have many limitations, such as low protein stability, rapid rate of cellular internalization, and relatively short half-life (for VEGF is 40 min under cellular conditions in vitro) [19, 24]. A way to bypass these obstacles is to use plasmid coding cytokines.

Based on previous studies and the fact that angiogenesis is a complicated process that requires many proangiogenic factors for proper vessel formation, we have developed bicistronic vectors carrying plasmid of the internal ribosome entry site pIRES/VEGF165/HGF encoding human VEGF165 and HGF, which is a novel therapy of CLI and DM based on dual gen construction. The aim of the present study was to assess the safety, feasibility, and clinical efficacy of intramuscular application of this bicistronic plasmid vectors in patients with CLI and DM. In addition, results of present study will be used to estimate the development of angiogenesis caused by expression of two angiogenic cytokines VEGF and HGF.

## Methods

### Plasmid DNA Preparation

**Fig. 1:**
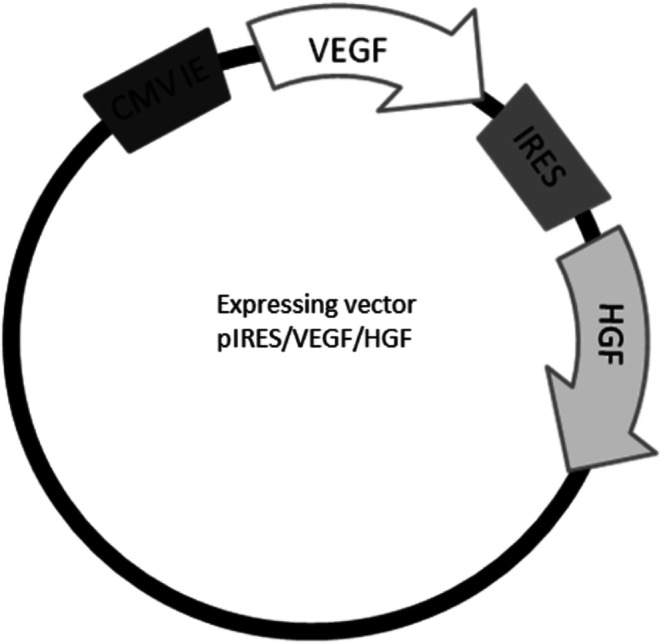
Schematic diagram of the plasmid structure

Bicistronic plasmid has been designed based on our previous research. We have used CMV promoter because it showed significant transfection efficiency in vitro [22]. We present the schematic diagram of the plasmid structure (Fig. Fig. 1).

Human HFG and VEGF165 cDNA were prepared as described previously [2]. Both cDNAs were cloned into pIRES bicistronic plasmid with the restriction enzymes. All plasmids pIRES/VEGF165/HGF prepared in this way were purified, dissolved and their apyrogenicity was confirmed. The whole process was already described in detail [2].

### Patient Cohort

The inclusion criteria for this study were (1) type 2 DM, (2) CLI, including rest pain and non-healing ischemic ulcers persisting for at least 12 weeks or gangrene, (3) resistance to conventional therapy of CLI for at least 4 weeks after hospitalization, (4) ankle-brachial index (ABI) of less than 0.4 in the affected limb and (5) NCR. Patients with the following conditions were excluded: severe retinopathy, end-stage renal disease, heart failure, angina pectoris, New York Heart Association (NYHA) Classification III or IV, liver dysfunction (grade B or C in the Child-Pugh classification), malignancy, history of malignancy, inability to stand or walk without help. Twenty-eight patients were enrolled in the study. They were randomized into two equal groups. Fourteen patients (ten men and four women aged from 58 to 81 years—average age 65.8) underwent bicistronic pIRES/VEGF165/HGF therapy—group 1. The remaining patients (nine men and five women aged from 40 to 85 years—average age 68.3) as control group did not receive plasmids—group 2. The duration of DM for all the patients ranged from 6.5 to 28 years. They required constant insulin administration every day with an average dose of 0.73 U/kg (range from 0.48 to 0.91 U/kg per day). The average haemoglobin A1c level was 8,7.1% (range from 6.3–12.1%). All patients from both groups were followed for 4 weeks under conventional conditions (antiplatelet and statin drugs, maintain normal glucose levels, physical training, wound debridement and broad-spectrum antibiotics if ulcers showed clinical signs of infection).

### Administration of Bicistronic Plasmid

Intramuscular injection of pIRES/VEGF165/HGF was performed into the ischemic lower limb above and below the knee level. Each patient received 4 mg of bicistronic plasmid. The injection sites to muscles were based on our previous study and literature data [15, 21]. The volume of each injection was 2.5 mL (approximately 80 injections, 2 cm deep into muscles of ischemic limb along each of the three crural arteries). The duration of injections did not exceed 1 h.

### Clinical Assessment

One, 4 and 12 weeks after treatment initiation, all patients were tested for haemoglobin, thrombocytes, leukocytes, C-reactive protein, creatinine and haemoglobin A1c levels. Heart rate, blood pressure and body temperature were also measured.

### Level of VEGF in Serum

Venous blood of patients in group 1 was taken from the upper limb to assess VEGF165 concentration. Test was performed before receiving plasmid injection, 7, 14, 28 and 90 days after. Serum was centrifuged, frozen, then evaluated using ELISA method.

### ABI

ABI was recorded 1 week before, 1 and 3 months after the treatment initiation in both groups of patients. It was calculated as the ratio of the lowest pressure from either the anterior or posterior tibial artery divided by the greatest brachial systolic pressure.

### Rest Pain

Rest pain was examined 1 week before, 1 and 3 months after the treatment initiation in both groups of patients. Pain assessment was evaluated using a self-administered visual analogue scale (VAS, 0–10).

### Computed Tomography Angiography

Computed tomography angiography (CTA) performed using a GE Medical Systems LightSpeed 16-slice device was used to evaluate the arterial supply of the lower limb in all patients. The first examination was carried out 1 week before the treatment initiation. The follow-up examination was performed 3 months after the initial test. CTA was performed after intravenous administration of 150 mL (ca 2 mL/kg b.w.) non-ionic agent.

### Statistical Analysis

Statistica 13.3 (StatSoft Polska) was used for statistical analysis. The following tests were used in the study: paired Chi-squared, Wilcoxon and Student’s *t* tests. Continuous variables were compared before and after therapy analysed by groups and time points. *P* < 0.05 was considered significant.

## Results

### Clinical Follow-Up

Intramuscular injections of bicistronic plasmid were administrated to 14 patients of group 1. The therapy was well tolerated and there were no major complications. Several minor complications occurred within 24 h following injections: tenderness at the injection sites (two patients) and fever (three patients). During our study, all the patients from both groups had no significant changes in laboratory parameters; none of them was hospitalized during follow-up. Some of the patients obtained surgical debridement of necrotic tissue. Two patients on group 1 and four patients in group 2 required limb amputation because of advanced CLI (necrosis, wide ulcerations and severe infection).

**Fig. 2:**
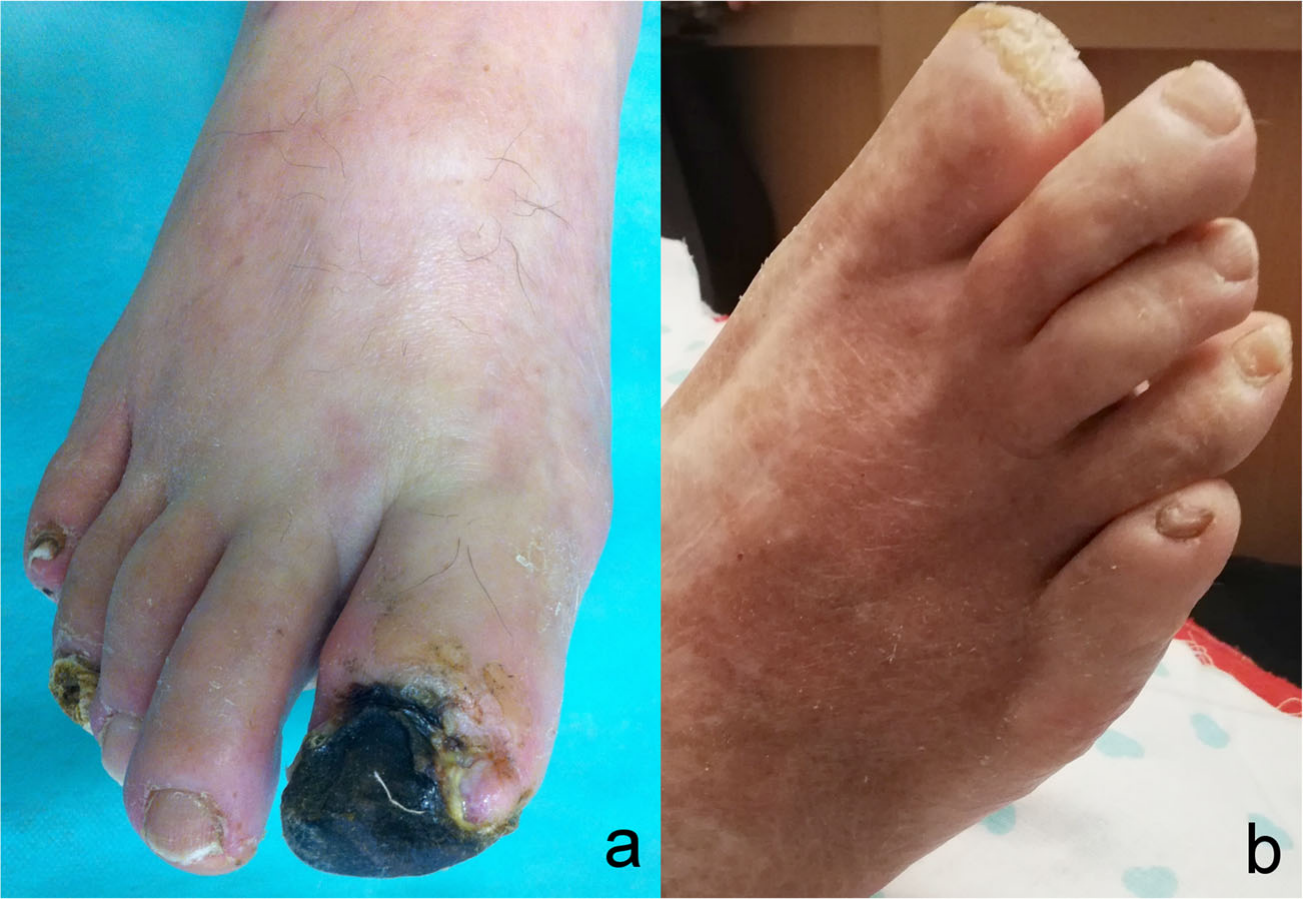
Clinical presentation; a: before plasmid administration; b: after 3 months from plasmid administration

All six patients were amputated below the knee. Comparing both groups, the wounds healed significantly better in group 1 (all patients while excluding amputated ones) than in group 2 (there were no complete healing). We present pictures of limb before and 3 months after bicistronic plasmid injections (Fig. 2).

### Level of VEGF in Serum

During our 90-day observation period, level of VEGF was measured in group 1, showing that fluctuations in cytokine VEGF levels occurred. Mean VEGF serum levels, as presented in Table 1, increased significantly from 235 ± 83 to 398 ± 78 pg/L (*p* < 0.001) 1 month after gene therapy. It was the highest mean serum level recorded during this study. However, the changes of VEGF levels were highly variable.

**Table 1:** Plasma level of VEGF (mean ± SD; *P* < 0.001)

Time of measurement	Before administration	7 days after administration	14 days after administration	28 days after administration	90 days after administration
Level of VEGF	235 ± 83	342 ± 85	391 ± 82	398 ± 78	395 ± 69

### Rest Pain (VAS)

We measured pain in all the patients (both groups) by VAS. There was significant decrease of pain in group 1: from 6.7 ± 1.4 to 1.3 ± 1.1 3 months after plasmid injections (*p* < 0.002). The best results concerned 12 patients from group 1, who obtained complete ulcer healing, while pain outcome in group 2 did not decrease significantly.

### Plasma Glucose Levels

Fasting plasma glucose level was adequate to guidelines of Polish Diabetes Association throughout observation and ranged between 6.5 and 8 mmol/L.

### ABI Results

The mean ABI increased significantly in group 1 from 0.28 ± 0.22 to 0.51 ± 0.29 (*p* < 0.001) 4 weeks after the administration of bicistronic plasmid. At the end of the study (after 3 months), the index increased significantly to 0.49 ± 0.30 (Table 2). In group 2, ABI results were as follows: 0.31 ± 0.27 before treatment, 0.36 ± 0.32 after 1 month, 0.32 ± 0.28 after 3 months. Results between groups changed significantly (*P* = 0.03). Six patients (two in group 1, four in group 2) did not have a chance to complete the ABI examination due to amputation.

**Table 2:** ABI results (mean ± SD; *P* = 0.03)

Time of measurement	One week before	One month after	Three months after
Group 1 (received plasmid)	0.28 ± 0.22	0.47 ± 0.29	0.51 ± 0.30
Group 2 (control group)	0.31 ± 0.27	0.36 ± 0.32	0.32 ± 0.28

### CTA

We performed CTA before and 90 days after plasmid administration. The radiologist, who evaluated CTA, did not know the patients and their treatment status. CTA documented typical findings of progressive peripheral atherosclerotic changes typical for diabetic microangiopathy in all 28 cases. These findings included segmental occlusive disease involving primarily the distal superficial femoral artery and/or the popliteal artery. We compared the results by calculating visible collateral vessels (Table 3). Three months after administration of bicistronic plasmid, formation of new collateral vessels was observed in all group 1 patients without amputation: from 106.7 ± 24.5 to 127.6 ± 27.6. It is statistically significant (*P* = 0.04) improvement compared with the group 1. Angiograms (Fig.s 3 and 4) showed qualitative evidence of improved distal flow after the gene.

**Table 3:** CTA results - number of visible collateral vessels (mean ± SD; *P* = 0.04)

Time of measurement	Before treatment	Three months after
Group 1 (received plasmid)	106.7 ± 24.5	127.6 ± 27.6
Group 2 (control group)	106.3 ± 22.6	112.6 ± 20.9

**Fig. 3:**
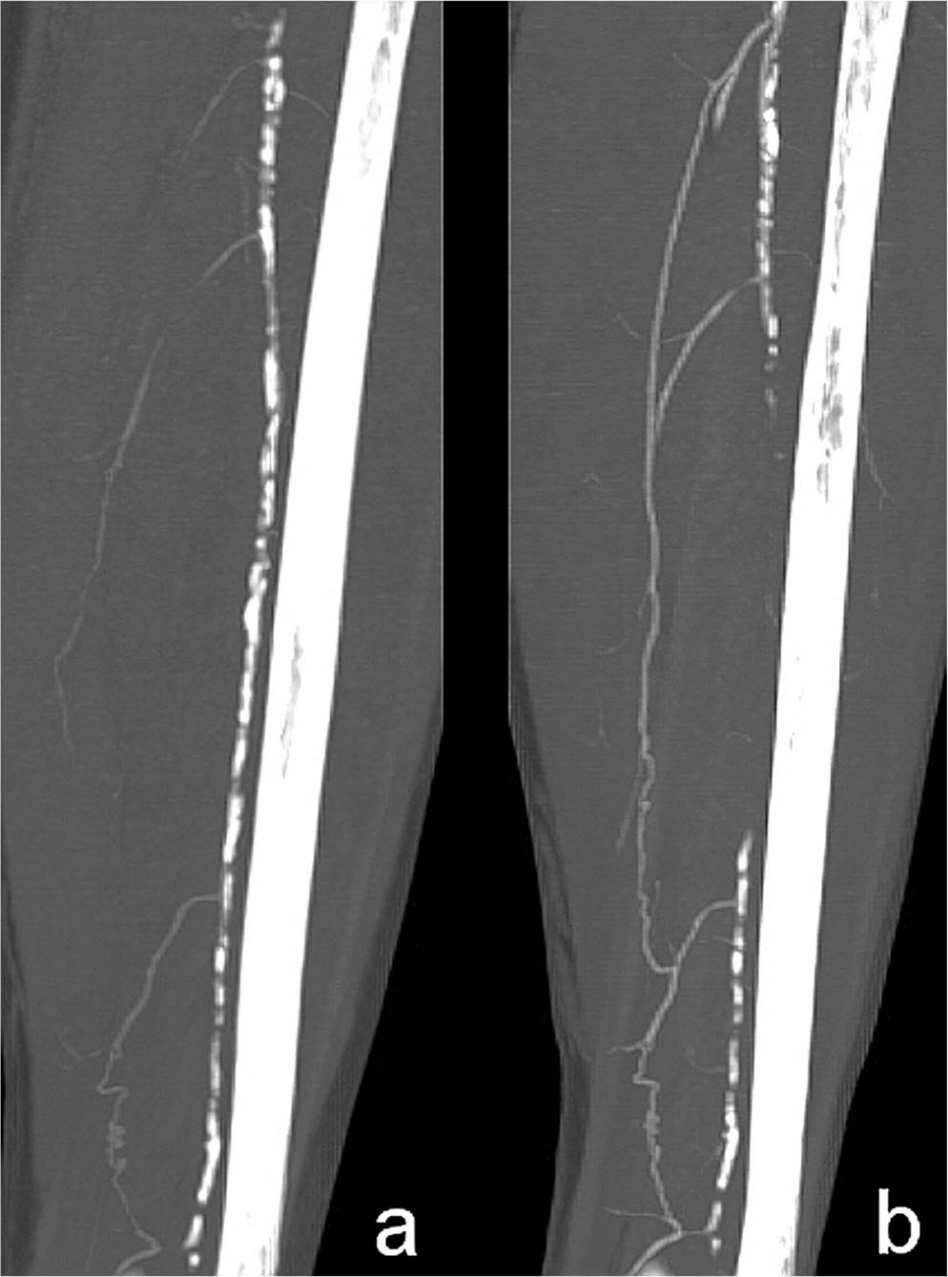
CTA angiograms – femoral longitudinal projection, shows the increase in length of collaterals; a: before plasmid administration; b: after 3 months from plasmid administration

**Fig. 4:**
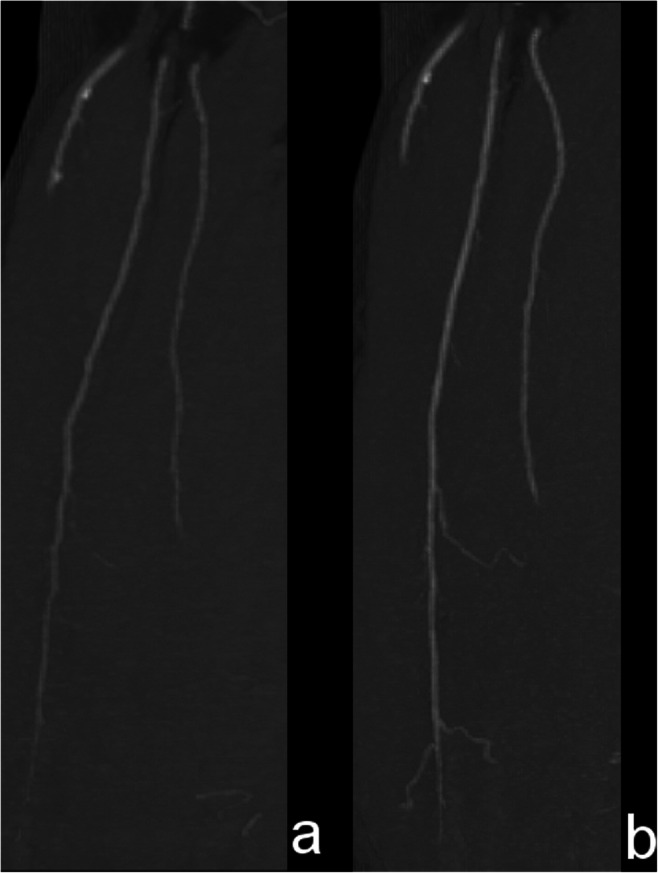
CTA angiograms – lower leg longitudinal projection, shows the increase in length of collaterals; a: before plasmid administration; b: after 3 months from plasmid administration

## Discussion

CLI is a life-limiting and life-threatening condition, which is mainly treated by risk factor modification and revascularization [15]. However, a large number of patients are NCR. In these cases, when pharmacological treatment is ineffective or insufficient, double VEGF/HGF gene therapy might be an attractive option. It is well known that VEGF, HGF, or FGF are key factors in the process of proliferation and revascularization [11]. We know that these molecules are produced in case of ischemia and their level depends on intensity of the process [26]. Our therapy leads to an increase in the level of proangiogenic factors in affected tissues. As alluded previously, there are studies showing that the release of HGF to extracellular matrix increases the activity of VEGF during neoangiogenesis [2, 20, 25]. Based on the evidence outlined above, we believe that double gene therapy has the potential to be more efficient than the single gene therapy.

This study examined the safety and efficacy of bicistronic gene therapy in patients suffering from CLI complicated by DM. There is a number of reports of using HGF plasmid, FGF or VEGF alone or combined with stem cells in treatment of CLI with good or moderate effect [18, 23]. There were attempts of using gene therapy based on intramuscular injections of VEGF or FGF plasmids in coronary heart disease with rather poor outcomes. To our knowledge, however, this is the first study describing the application of pIRES/VEGF165/HGF therapy using bicistronic plasmid administration in patients with CLI and DM. The treatment regimen described in this study displayed high efficacy with little side effects which is in line with previously published studies of similar therapeutic interventions. The ABI improved and rest pain decreased significantly after 3 months. The CTA demonstrated considerable improvement of vascularization. Additionally, no serious complications were observed. Despite the positive findings of this study, we suggest that further studies should focus on maximizing the efficacy of double gene therapy by improving technical aspects of the procedure. Preprocedural mapping of areas with vascular deprivation with adequate imaging and image guidance for gene delivery directly to the muscular site of interest, which is missing in many of the gene transfer studies, would certainly limit the unintended delivery of therapeutic agents to the fascia or subcutaneous tissue [15]. To address this issue, in order to maximize the efficacy of our treatment, we were giving intramuscular injections near the major affected artery. We evaluated the arteries before therapy using CTA and in physical examination based on common knowledge from angiostomy. In fact, the injection technique utilized in this study was similar to that previously used by Amman et al. with intramuscular injection of bone marrow mononuclear cells in patients with CLI [1].

The study described above, despite showing positive results, also has a number of limitations. The control group was not placebo-controlled or double blind. ABI which we used to assess the efficacy of the therapy might not be a sensitive test in DM patients. The VAS scale used in this study can also be subjective. Due to financial restrictions, we were not able to verify pIRES/VEGF165/HGF in human cell lines in vitro. For the same reason, we could measure only VEGF plasma level in therapeutic group, not both: VEGF and HGF in both groups.

Despite these limitations, the treatment regimen described in this study led to a highly statistically significant improvement in outcomes in patients and has considerably improved their quality of life. Based on the results of this study, we suggest that the pIRES/VEGF165/HGF therapy using bicistronic plasmid administration is a safe and effective method of treating patients with both CLI and DM. The fact that patients suffering from both CLI and DM often present with additional comorbidities and are likely to undergo procedures such as leg amputations makes the findings of our study all the more important. It should be noted that the effectiveness of this treatment is highly dependent on its early application in the disease progression. Further clinical studies with larger sample sizes should be performed to confirm the efficacy and safety of the pIRES/VEGF165/HGF therapy in CLI and DM.

## Abbreviations

ABI: Ankle-brachial index
CLI: Critical limb ischaemia
CTA: Computed tomography angiography
DM: Diabetes mellitus
FGF: Fibroblast growth factor
HGF: *Hepatocyte growth factor*
NCR: Not candidate for revascularization
NYHA: New York Heart Association
PAD: Peripheral arterial disease
VAS: Visual analogue scale
VEGF: Vascular endothelial growth factor
